# Modulation of Short-Latency Afferent Inhibition Depends on Digit and Task-Relevance

**DOI:** 10.1371/journal.pone.0104807

**Published:** 2014-08-13

**Authors:** Michael J. Asmussen, Christopher M. Zapallow, Mark F. Jacobs, Kevin G. H. Lee, Philemon Tsang, Aimee J. Nelson

**Affiliations:** Department of Kinesiology, McMaster University, Hamilton, Canada; University Medical Center Goettingen, Germany

## Abstract

Short-latency afferent inhibition (SAI) occurs when a single transcranial magnetic stimulation (TMS) pulse delivered over the primary motor cortex is preceded by peripheral electrical nerve stimulation at a short inter-stimulus interval (∼20–28 ms). SAI has been extensively examined at rest, but few studies have examined how this circuit functions in the context of performing a motor task and if this circuit may contribute to surround inhibition. The present study investigated SAI in a muscle involved versus uninvolved in a motor task and specifically during three pre-movement phases; two movement preparation phases between a “warning” and “go” cue and one movement initiation phase between a “go” cue and EMG onset. SAI was tested in the first dorsal interosseous (FDI) and abductor digiti minimi (ADM) muscles in twelve individuals. In a second experiment, the origin of SAI modulation was investigated by measuring H-reflex amplitudes from FDI and ADM during the motor task. The data indicate that changes in SAI occurred predominantly in the movement initiation phase during which SAI modulation depended on the specific digit involved. Specifically, the greatest reduction in SAI occurred when FDI was involved in the task. In contrast, these effects were not present in ADM. Changes in SAI were primarily mediated via supraspinal mechanisms during movement preparation, while both supraspinal and spinal mechanisms contributed to SAI reduction during movement initiation.

## Introduction

Short-latency afferent inhibition (SAI) occurs when a single transcranial magnetic stimulation (TMS) pulse over the primary motor cortex (M1) is preceded by peripheral electrical nerve stimulation at a short inter-stimulus interval (i.e., ∼20–28 ms) such that the corticospinal output to the targeted hand muscle is reduced [1], [2]. SAI may contribute to another circuit known as surround inhibition that is important to performing individual finger movement. Surround inhibition is a powerful neurophysiological mechanism that focuses neural activity by inhibiting areas surrounding the intended neural response. This mechanism has been observed in the visual [3], somatosensory [4], and motor systems [5]. Particularly, surround inhibition in the motor system may be a mechanism that allows for precise selective movements by enhancing neural activity for muscles performing a task, while inhibiting neural activity for those muscles uninvolved in the task.

The functional significance of SAI to hand control remains largely unknown yet an active muscle can modify the magnitude of SAI [6], [7]. We and others have shown that SAI is reduced during both the onset of muscle activity [6], [8], [9] and during sustained muscle contraction [6], [7]. Specifically, we observed reductions in SAI as early as movement preparation between an auditory “warning” and “go” cue and these reductions are likely cortically or sub-cortically mediated [6]. Previous work [8] has suggested that SAI may contribute to surround inhibition, particularly during EMG onset. However, a number of questions regarding the functional significance of SAI remain unexplored. First, how is SAI modified when the muscle is involved versus uninvolved in the task? [8], [9]. Second, does the modulation of SAI depend on the specific digit (i.e., digit 2 versus digit 5)? Compared to the 5^th^ digit, the 2^nd^ digit may play a greater role in grasping and has a larger cortical representation that could lead to changes in SAI [10]–[13]. Third, how does SAI act before EMG onset and before movement initiation? It may be that the SAI circuit is involved in focussing neural activity differently depending on the specific digit that is or is uninvolved in the task.

The purpose of this study was to determine whether SAI is modulated during movement preparation (i.e., between a “warning” and “go” cue) and movement initiation (i.e., between a “go” cue and onset of muscle activity) when a muscle is involved or uninvolved in a finger flexion task. SAI was measured in the first dorsal interosseous (FDI) and abductor digiti minimi (ADM) to represent muscles controlling the 2^nd^ and 5^th^ digit, respectively, which contribute differently to the overall functional capacity of the hand. In a second experiment, spinal excitability via Hoffman reflexes (H-reflexes) were measured in FDI and ADM during the same motor task.

## Methods

### Ethics Statement

This study was approved by the Office of Research Ethics at McMaster University and conformed to the *Declaration of Helsinki*. Written informed consent was obtained from all participants in the study.

### Participants

Twelve healthy individuals (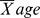 = 20, SD = 2, 5 females) participated in Experiment 1 and of those, ten subjects (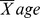 = 20.1, SD = 2.1, 6 males) participated in Experiment 2. All participants were deemed to be right handed determined using a modified version of the Edinburgh Handedness Inventory [14]. All participants were screened for any contra-indicators of TMS (i.e., no intake of benzodiazepines).

### Electromyography (EMG)

Surface Ag/AgCl EMG electrodes were placed on the FDI and ADM muscles of the right and left hands in a muscle belly-tendon montage. The right hand was engaged in the task while the left hand remained relaxed throughout the experiments. The analog signal from the electrodes was amplified with a gain of 1000, band-pass filtered between 20 and 2500 Hz (Intronix Technologies Corporation Model 2024F, Bolton, Canada) and sampled at a frequency of 5000 Hz using an analog-to-digital interface (Power 1401, Cambridge Electronic Design, Cambridge, UK). The EMG electrodes were used to record the electrical signal to the muscles and the recorded signal was used to measure the peak-to-peak amplitude of the MEP elicited in the FDI and ADM of the right hand and ongoing muscle activity in the left hand. Analysis was completed off-line using Signal software (version 5.07, Cambridge Electronic Design, Cambridge, UK).

### Peripheral Nerve Stimulation

Peripheral nerve stimulation was achieved with 200 µs square wave pulses delivered using Grass SD9 Telefactor stimulators (Grass Technologies, West Warwick, USA). The digital nerves of the 2^nd^ and 5^th^ digits were stimulated using ring electrodes with the cathode proximal to the anode and positioned around the proximal and intermediate phalynx. Digital nerves were stimulated at ∼3 times perceptual threshold, an intensity shown to evoke SAI at rest [1]. To elicit H-reflexes, the ulnar nerve was stimulated (1 ms square wave pulse) at the wrist approximately 8 cm proximal to the thenar muscles of the right hand. The intensity of ulnar nerve stimulation was set to elicit M-waves of 10% of the direct maximal muscular response (M-wave_max_) in FDI or ADM. This intensity was used to ensure the H-reflex recorded was on the ascending portion of the H-reflex recruitment curve [15].

### Transcranial Magnetic Stimulation (TMS)

TMS was delivered using two custom built 50 mm diameter figure-of-eight branding coils connected to two Magstim 200^2^ stimulators (Magstim, Whitland, UK). Coil position and orientation was monitored throughout the experiment using Brainsight Neuronavigation (Rogue Research, Montreal, Canada) with optical sensors placed on the coil and the participant. The coil was oriented at 45° in relation to the parasagittal plane to induce a posterior-lateral to anterior-medial current in the cortex and preferentially activate corticospinal neurons trans-synaptically [16]. One TMS coil delivered a monophasic pulse over M1 in the optimal location to elicit MEPs in the FDI muscle of the right hand, while a separate coil delivered a monophasic pulse over the optimal location to elicit MEPs in the ADM muscle of the right hand. Only one coil was placed on the scalp at any time during data collection. The optimal hotspot for each muscle was obtained separately and was determined by the position of the coil that produced a MEP ∼1 mV at the lowest percentage of maximal stimulator output (%MSO). The hotspot for FDI was first identified and subsequently the ADM hotspot was located. The group-averaged difference between ADM hotspot in relation to the FDI hotspot was 3 mm medial and 7 mm posterior.

### Behavioural task

A similar behavioural task was performed in Experiments 1 and 2. At the beginning of the set-up, participants were seated with their right arm relaxed with their shoulder abducted ∼20° and elbow flexed at ∼90°. In this position, participants voluntarily flexed their finger at the metacarpophalangeal joint maximally against a load cell (Trandsucer Techniques, model THA-50-Q load cell, Temecula, USA). This measure was completed for the 2^nd^ and 5^th^ digits separately. Participants then practiced performing a phasic isometric finger flexion to 5% of their maximum force (F_max_) for their 2^nd^ and 5^th^ digit (5% F_max_), separately, using visual feedback of their force displayed on an oscilloscope. The 5% F_max_ was the force requirement for the behavioural task.

**Figure 1:**
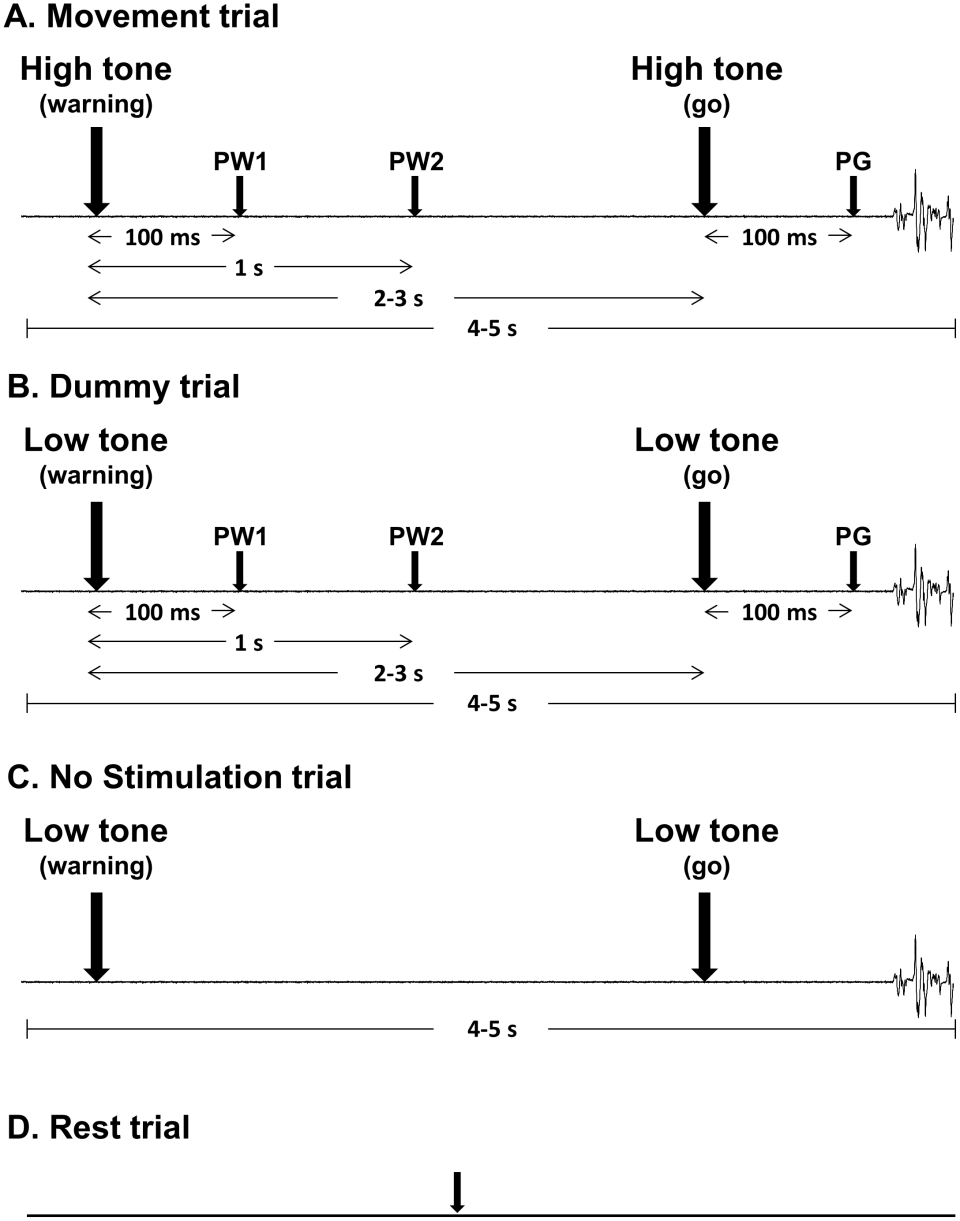
Timeline of the three trial types in a block and the rest trial. The ‘Warning’ and ‘Go’ represent the auditory ‘warning’ and ‘go’ cue, respectively. The small arrows pointing down indicate when SAI was tested. Before the experiment, the researcher defined whether the low and high tone meant move 2^nd^ or 5^th^ digit, respectively. The movement trial indicates trials that were used for analysis, while the dummy trial indicates trials that were not used for analysis. In this figure, 2^nd^ digit movement was being analyzed and the high tone indicates the movement condition, while the low tone would inform the participant to perform the 5^th^ digit movement and would serve as the dummy trial. ‘100 ms’ on the left side of the timeline represents the time between the ‘warning’ cue and when SAI was tested in the post-warning 1 phase (PW1). ‘1 s’ represents the time between the ‘warning’ cue and when SAI was tested in the post-warning 2 phase (PW2). ‘100 ms’ on the right side of the timeline represents the time between ‘go’ cue and when SAI was tested in the post-go phase (PG). ‘2–3 s’ is the varied interval between the ‘warning’ and ‘go’ cue, while the ‘4–5 s’ indicate the varied length of the trial. In the “no stimulation” condition, neither TMS nor nerve stimulation was delivered, but the participant still completed the trial with the ‘warning’ and ‘go’ cue present. The rest trial, SAI was tested, but no auditory cues were given and the participant did not complete the movement.

Each trial consisted of an auditory tone that served as the “warning” cue followed 2 to 3 seconds later by a second auditory tone that served as the “go” cue (*Figure 1*). Upon hearing the “go” cue, participants flexed their 2^nd^ or 5^th^ digit to 5% F_max_ against a load cell and released the contraction once 5% F_max_ was achieved (i.e., a phasic contraction). The voltage from the load cell was passed through a strain gage amplifier (Futek model CSG110-FSH03546, Thornhill, Canada) and the online force level achieved by the 2^nd^ or 5^th^ digit was displayed on an oscilloscope as a bright line. Subjects were required to position one line which represented their current force level over another line that marked the 5% F_max_ for that particular digit. In a single block, there were three trial types. One trial type would test the movement condition that was used for analysis (ex., move 2^nd^ digit, SAI in FDI) and, in the example in *Figure 1*, the high tone would be used for both the “warning” and “go” cue (*Figure 1A*). The second trial type was a dummy trial that the participant would perform the other type movement. If the 2^nd^ digit movement was the condition for analysis, the low tone was used in the dummy trial for both the “warning” and “go” cue and would indicate 5^th^ digit movement (*Figure 1B*). If the block was testing 5^th^ digit movement (i.e., move 5^th^ digit, SAI in FDI), the trials for analysis would have the low tone serve as the cues, while the high tone would serve as the cues for the dummy trials and require the participant to move the 2^nd^ digit. The “go” cue was always congruent with the tone of the “warning” cue. For example, if the warning cue's tone indicated 2^nd^ digit movement, the go cue would be the same tone and indicate to move the 2^nd^ digit. The researcher informed the participant the meaning of the high versus low tone before the experiment. The no stimulation trial would have tones present and the participant would perform the movement, but no stimulation was given (*Figure 1C*). The rest trial was performed in a separate block and the timeline is depicted in *Figure 1D*.

### Experiment 1: SAI as a function of task and phase

SAI was investigated in FDI and ADM by placing the coil on the motor hot spot for each respective muscle. To elicit SAI in FDI or ADM, stimulation was applied to the digital nerve of the 2^nd^ or 5^th^ digit, respectively. SAI in these muscles was investigated during three pre-movement phases prior to EMG onset such that a single TMS pulse was delivered either 100 ms after the “warning” cue in the post-warning 1 phase (PW1) (*Figure 1*), 1000 ms after the “warning” cue in the post-warning 2 phase (PW2), or 100 ms after the “go” cue in the post-go phase (PG). Any trial in which participants anticipated the “go” cue (EMG at or before 100 ms) was rejected offline and not included in the analysis. SAI was tested in each muscle (FDI, ADM) and at each time point (PW1, PW2, PG), while the participant was in the context of performing either 2^nd^ or 5^th^ digit movement dictated by the tone frequency. Twelve conditions were tested in total: 3 pre-movement phases (PW1, PW2, PG) x 2 types of movement (2^nd^, 5^th^ digit flexion) x SAI in 2 muscles (ADM, FDI). Twenty trials were completed for each condition whereby ten trials delivered a single TMS pulse only (i.e., unconditioned MEP) and the other ten delivered stimulation to the appropriate digital nerve 25 ms prior to the TMS pulse to evoke SAI (i.e., conditioned MEP) [7]. The order of conditioned and unconditioned MEPs was randomized. For FDI and ADM, the stimulator output was adjusted to elicit an unconditioned MEP of ∼1 mV from a single TMS pulse in each of PW1, PW2 and PG, while the participant was preparing to perform either 2^nd^ or 5^th^ digit movement. The unconditioned MEP intensity was determined before each block and when participants practiced two to four trials of that particular condition. During these practice trials, the TMS intensity to elicit an ∼1 mV MEP response was determined and used for that particular block of trials. In addition, online minor adjustments to the TS intensity were performed if necessary to maintain the unconditioned MEP amplitude at ∼1 mV. The reason for adjusting the stimulator output for each condition is that the TMS intensity to elicit an MEP of ∼1 mV in the targeted muscle might be different for preparation of 2^nd^ versus 5^th^ digit movement. Further, when the muscle is involved or uninvolved in the task, the MEP could be increased or reduced, respectively, and the differing amplitude of the unconditioned MEP can affect the degree of SAI [7]. Therefore, the group-averaged %MSO for each condition is presented in Table 1 and Table 2 displays the group-averaged unconditioned MEP amplitude (i.e., TS alone) for each particular condition. Each of the 12 conditions were performed in separate blocks. The inter-trial interval varied between 4 and 5 seconds. Additionally in a block, no stimulation trials (i.e., no TMS or nerve stimulation) and dummy trials (i.e., required to move 5^th^ digit when SAI is being tested during a 2^nd^ digit movement) were included to avoid the participant predicting the trial type being tested in the block and prevent any anticipation effects of TMS or nerve stimulation. The block order was randomized across participants. In addition to the pre-movement trials, rest trials were performed whereby participants were required to relax their hand completely. Twenty resting trials were completed; ten unconditioned and ten conditioned MEPs, and split up into two blocks for each muscle (i.e., 4 blocks total). The rest blocks were either performed before and in the middle of the testing blocks or in the middle and the end of the testing blocks.

**Table 1:** Percentage of MSO to obtain ∼1 mV MEP in each condition.

	Rest	Move 2^nd^ digit			Move 5^th^ digit		
		PW1	PW2	PG	PW1	PW2	PG
FDI	51±3	52±3	51±3	47±4	53±3	53±4	55±4
ADM	60±3	61±4	61±5	61±4	61±5	60±4	53±4

Means (% MSO) followed by standard error are presented.

**Table 2:** Unconditioned MEP size for FDI and ADM during all conditions.

	Rest	Move 2^nd^ digit			Move 5^th^ digit		
		PW1	PW2	PG	PW1	PW2	PG
FDI	1.27±0.08	1.20±0.10	1.46±0.17	1.11±0.17	1.39±0.07	1.05±0.07	0.88±0.09
ADM	0.99±0.08	1.24±0.12	1.26±0.15	1.11±0.13	1.09±0.08	1.03±0.11	1.34±0.21

Mean MEP amplitude (mV) followed by standard error are presented.

For all pre-movement phases and rest trials, the experimenter rejected any trials offline whereby EMG had a peak-to-peak amplitude >20 µV over the resting background EMG signal 200 ms before the TMS pulse, similar to previous work [9]. If crossing this threshold was indicated during a trial, it was rejected online and the trial was repeated. If the threshold of EMG activity could not be detected online, the trial was rejected offline during data analysis. On average, 1.75 trials out of the total 20 trials testing a particular condition were rejected offline.

### Experiment 2: H-reflex as a function of task and phase

H-reflexes were used to investigate whether SAI modulation may occur via spinal and/or supraspinal mechanisms [17]. The H-reflex measure was used to determine if spinal excitability changes as a function of phase, muscle and task involvement. H-reflexes were obtained in FDI or ADM by having the participants produce a light voluntary contraction with their 2^nd^ or 5^th^ digit, respectively. This stimulus duration and light voluntary contraction was implemented because an H-reflex is more readily obtained in FDI and ADM when they are slightly active [18]. Participants performed the same behavioural task as in Experiment 1 (12 testing conditions with dummy and no stimulation trials and one rest condition) with one exception for all conditions. The participant maintained a light voluntary contraction in the digit that the targeted muscle for the H-reflex was actively involved in (e.g., 2^nd^ digit for FDI or 5^th^ digit for ADM H-reflex) and increased the force by ∼5% in response to the ‘Go’ cue. The force requirement during the task was the same for 2^nd^ and 5^th^ digit.

### Statistical analyses

SAI was calculated as the ratio of the conditioned MEP to the unconditioned MEP (
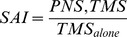
). Spinal excitability was defined with the following formula using the peak-to-peak amplitude of the H-reflex and M-wave: (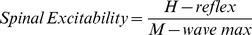). A three-way repeated measures ANOVA with factors PHASE (3 levels: PW1, PW2, PG), MUSCLE (2 levels: FDI, ADM), and MOVEMENT TYPE (2 levels: 2^nd^, 5^th^ digit) was conducted for SAI (Experiment 1) and spinal excitability (Experiment 2). For both ANOVAs the dependent measures of SAI and spinal excitability were normalized to the measure at rest (i.e., 
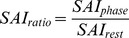
, 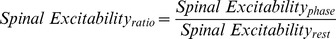). This normalization allows for the comparison of increases or decreases of SAI or spinal excitability between muscles (e.g., SAI reduction in FDI in relation to SAI reduction in ADM). Means less than 1 indicate increased SAI (or decreased spinal excitability) in relation to rest and means greater than 1 indicate reduced SAI (or increased spinal excitability) in relation to rest. Tukey's post-hoc analysis was performed if a significant effect was found.

Second, we tested whether there was a relationship between unconditioned MEP amplitude and degree of SAI. The reason for this analysis was to ensure that the unconditioned MEP amplitude did not affect the degree of SAI, as larger MEP amplitude could potentially yield reduced SAI [7]. Pearson's product moment correlation coefficient between the unconditioned MEP amplitude (i.e., TMS_alone_) and the degree of SAI (
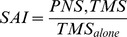
) in each muscle during both 2^nd^ and 5^th^ digit movement [6]. Last, we conducted a three-way repeated measures ANOVA with factors PHASE (3 levels: PW1, PW2, PG), MUSCLE (2 levels: FDI, ADM), and MOVEMENT TYPE (2 levels: 2^nd^, 5^th^ digit) on the unconditioned MEP amplitude. For all statistical tests, the alpha level was set at *p*≤0.05. Sphericity was tested and when this assumption was violated the Greenhouse Geisser correction was implemented and the adjusted degrees of freedom were reported.

## Results

### Experiment 1: SAI as a function of task and phase

Experiment 1 examined whether SAI was dependent on the specific digit and its involvement in the task being performed. The group mean F_max_ for the 2^nd^ digit was 30.9 N±11.5 and 18.5±6.9 for the 5^th^ digit similar to previous research [6]. Although the nerve stimulation was delivered at the same intensity in relation to perceptual threshold, SAI at rest was greater in FDI compared to ADM (*t*_(11)_ = 3.04, *p* = 0.011).

**Figure 2:**
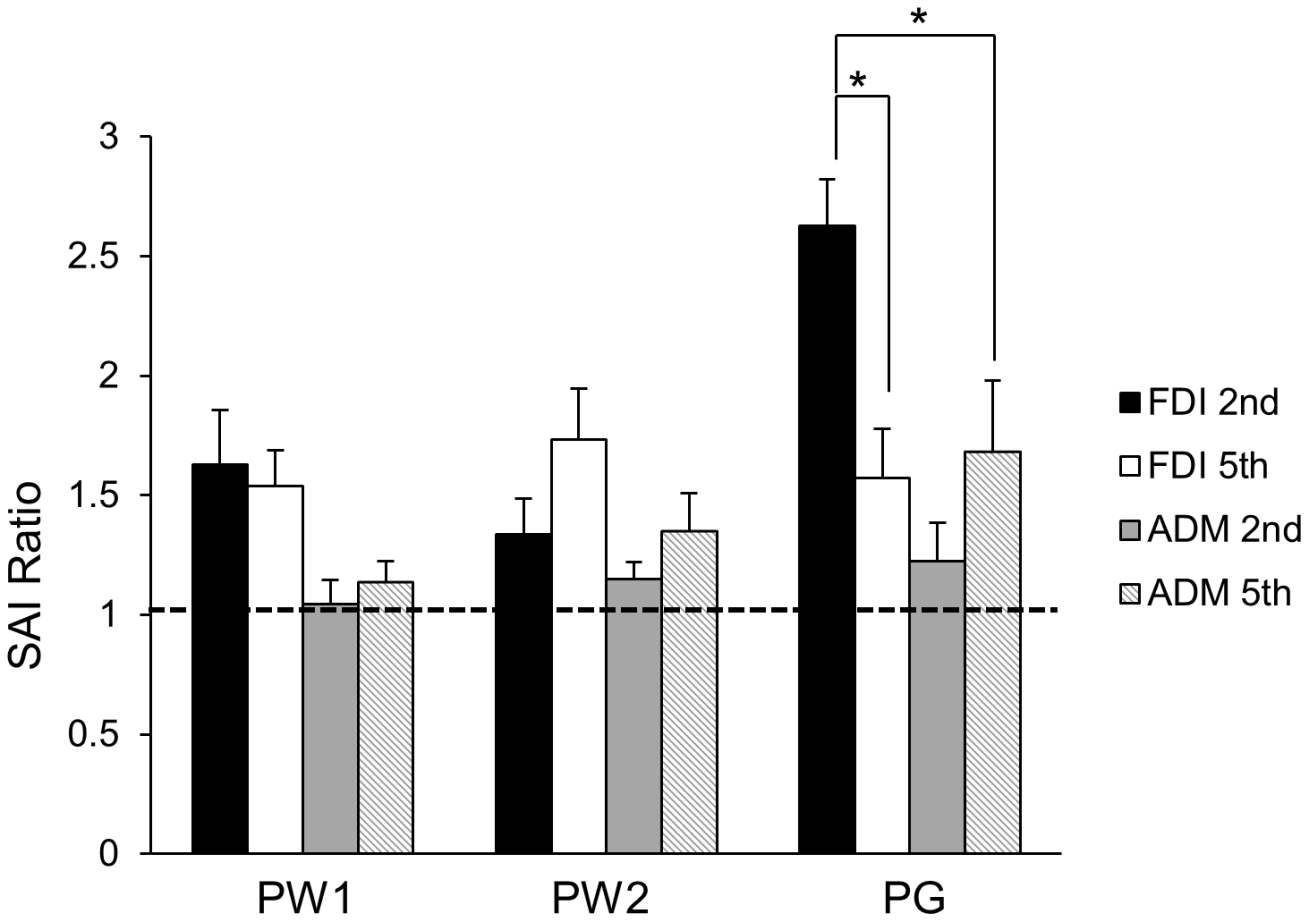
Differences in SAI across the three pre-movement phases. Group-averaged SAI ratio data (with standard error of the mean) for each pre-movement time point (i.e., PW1, PW2, PG) and muscle (FDI, ADM). Values greater than 1 indicate a reduction in SAI, while values less than 1 indicate an increase in SAI. An asterisk over a bar connecting two different conditions indicates significant differences. Significant differences were tested at *p*≤0.05.

**Figure 3:**
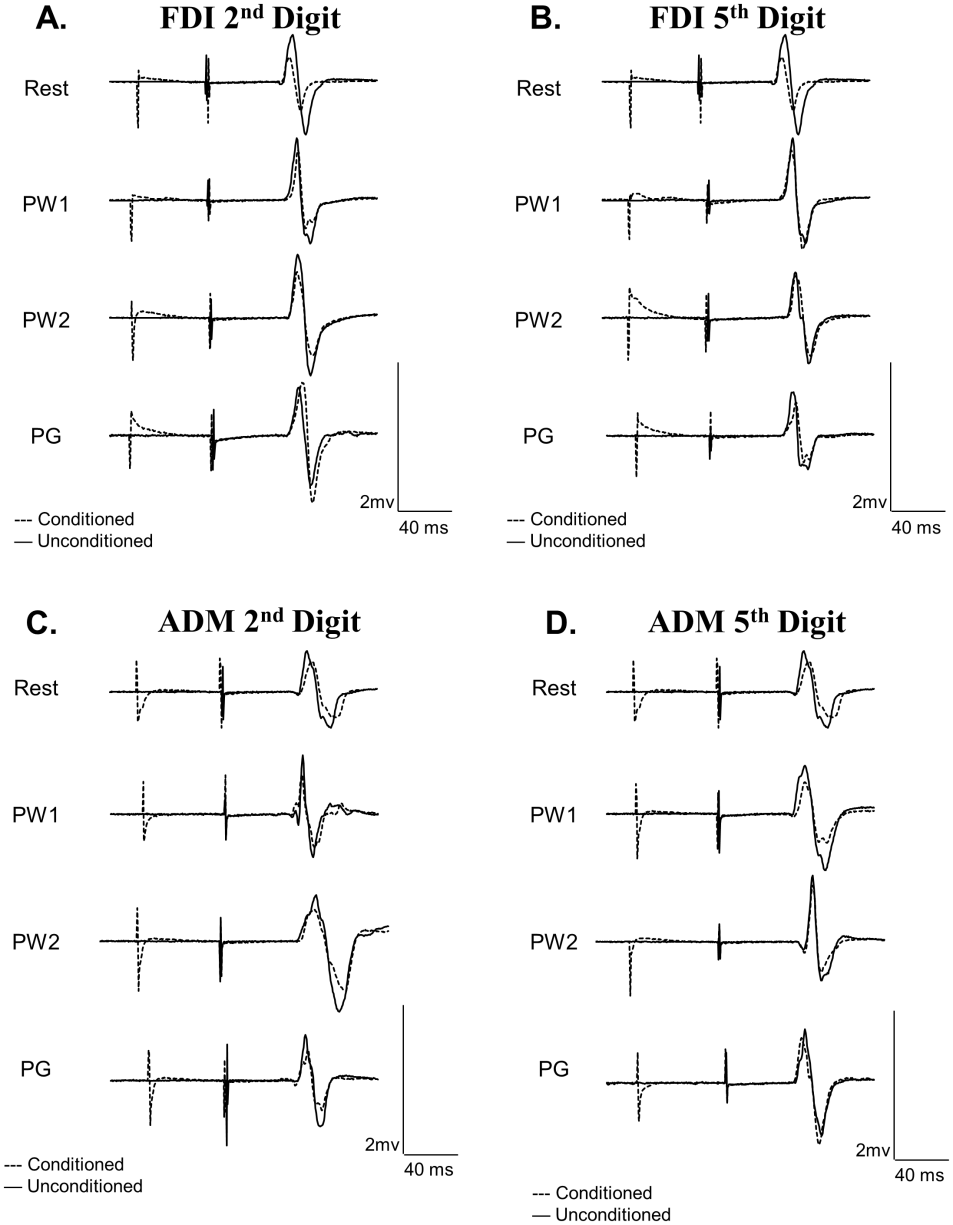
Raw traces of conditioned and unconditioned MEPs for FDI and ADM. Each trace is an individual trial that is representative of the group-averaged data. **A**: Raw EMG traces of SAI in FDI during preparation for 2^nd^ digit movement. **B**: Raw EMG traces of SAI in FDI during preparation for 5^th^ digit movement. **C**: Raw EMG traces of SAI in ADM during preparation for 2^nd^ digit movement. **D**: Raw EMG traces of SAI in FDI during preparation for 5^th^ digit movement.

*Figure 2* displays the group-averaged SAI ratio (with standard error of the mean) during the pre-movement phases (PW1, PW2, PG) for each muscle (FDI, ADM) and each task (involved, uninvolved). The raw traces of the unconditioned and conditioned MEP for each movement condition are presented in *Figure 3*. The repeated measures ANOVA revealed a significant three-way interaction between PHASE, MOVEMENT TYPE, and MUSCLE (*F*_(1.3,14.4)_ = 4.803, *p* = 0.037), a PHASE by MOVEMENT interaction (*F*_(2,22)_ = 5.238, *p* = 0.024), and main effect of PHASE (*F*_(2,22)_ = 4.069, *p* = 0.031) and MUSCLE (*F*_(1,11)_ = 19.520, *p* = 0.001). Post-hoc Tukey's test revealed two significant differences in the PG phase, but no significant differences in PW1 or PW2. First, SAI was reduced in FDI when it was involved versus uninvolved in the task during movement initiation (*p*<0.05). There were no differences of SAI in ADM when it was involved versus uninvolved in the task. Second, SAI in FDI was significantly reduced compared to ADM when each muscle was involved in the task (*p*<0.05). There were no differences in SAI between FDI and ADM when there were uninvolved in the task. These data indicate that SAI reduction are task and muscle specific. Table 3 indicates the group means (with standard error of the mean) for the SAI ratio data during rest and the pre-movement phases.

**Table 3:** SAI data and SAI ratio for FDI and ADM during all conditions.

	SAI Data							SAI Ratio Data					
Muscle	Rest	Move 2^nd^ digit			Move 5^th^ digit			Move 2^nd^ digit			Move 5^th^ digit		
		PW1	PW2	PG	PW1	PW2	PG	PW1	PW2	PG	PW1	PW2	PG
FDI	0.51±0.053	0.80±0.126	0.66±0.087	1.32±0.233	0.78±0.104	0.87±0.122	0.74±0.064	1.63±0.229	1.34±0.195	2.63±0.374	1.54±0.152	1.73±0.211	1.57±0.208
ADM	0.68±0.051	0.68±0.06	0.77±0.065	0.76±0.057	0.75±0.072	0.88±0.105	1.14±0.267	1.05±0.101	1.15±0.068	1.22±0.163	1.13±0.089	1.35±0.161	1.68±0.301

Means for SAI data 
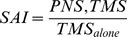
 and SAI ratio data 
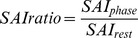
. HSD critical for a significance of 0.05 was calculated as 0.81 for comparing amongst SAI ratio data.

There was no significant correlation between the unconditioned MEP amplitude (i.e., TMS alone) and the magnitude of SAI (
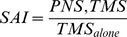
) in FDI during 2^nd^ digit movement (*r* = −0.176, *p* = 0.23), FDI during 5^th^ digit movement (*r* = −0.019, *p* = 0.896), ADM during 2^nd^ digit movement (*r* = −0.108, *p* = 0.466), nor ADM during 5^th^ digit movement (*r* = −0.148, *p* = 0.315), indicating that the changes in SAI were due to the movement phase and not MEP amplitude for every muscle in each type of movement. For the unconditioned MEP amplitude, a three-way interaction was revealed (*F*_(1.3, 14.8)_ = 7.243, *p* = 0.012). We analyzed differences at each movement phase as completed in the ANOVA for SAI. Post-hoc Tukey's did not reveal any significant differences at each movement phase (i.e., PW1, PW2, PG).

### Experiment 2: H-reflex as a function of task and phase

**Figure 4:**
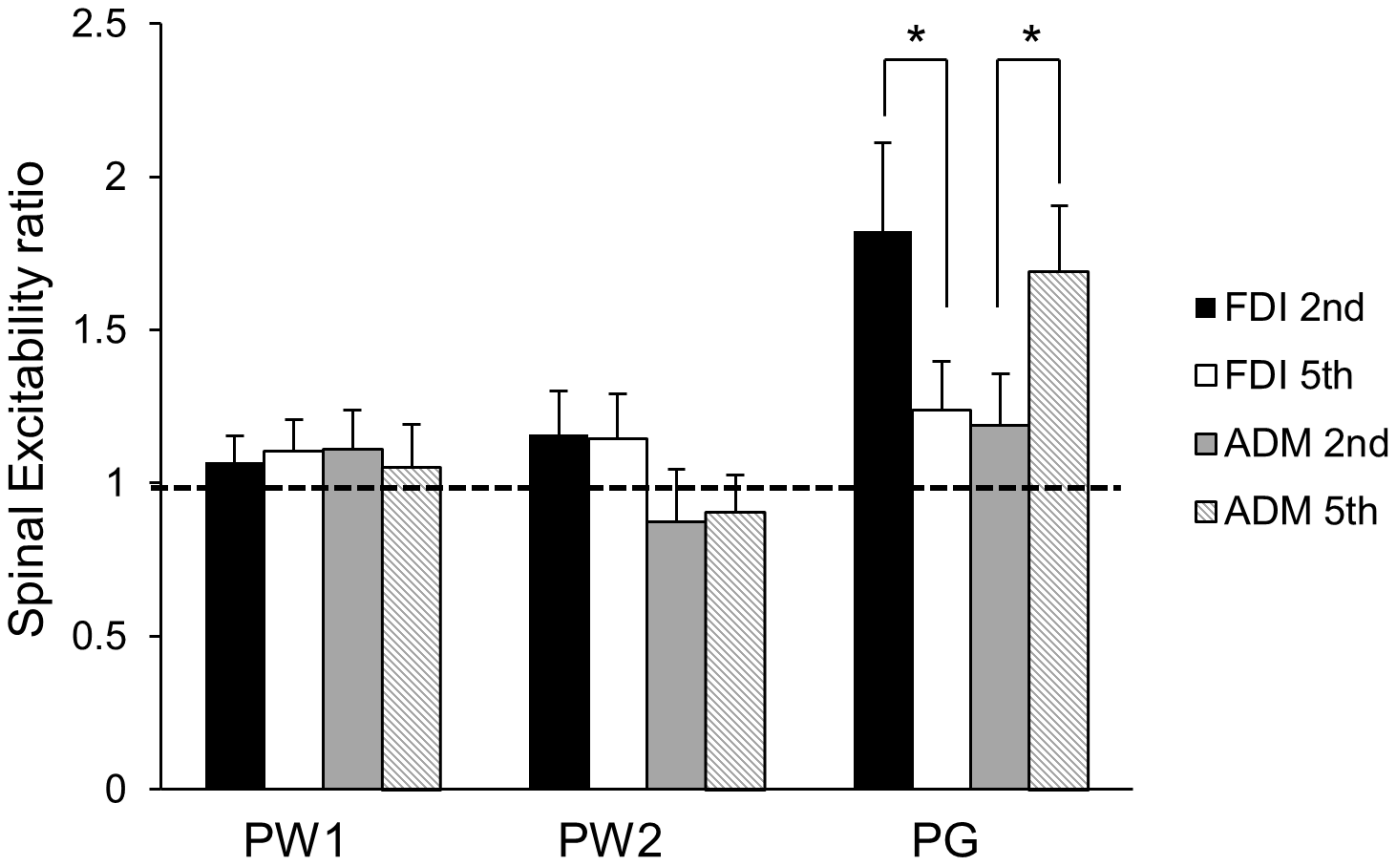
Differences in spinal excitability across the three pre-movement phases. Group-averaged spinal excitability data (with standard error of the mean) for each pre-movement time point (i.e., PW1, PW2, PG) and muscle (FDI, ADM). Values greater than 1 indicate an increase in spinal excitability, while values less than 1 indicate decreases in spinal excitability. An asterisk over a bar connecting two different conditions indicates significant differences. Significant differences were tested at *p*≤0.05.

**Figure 5:**
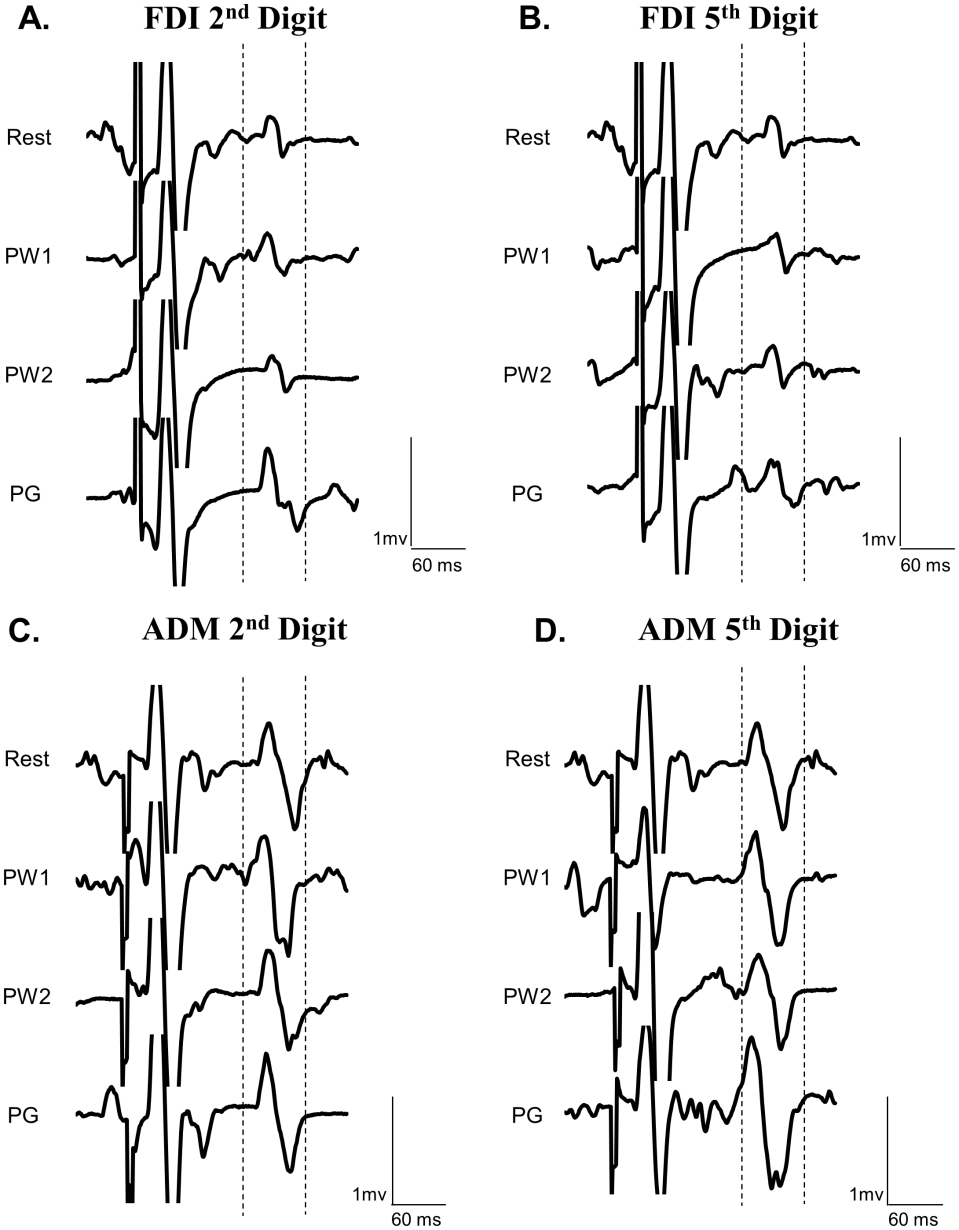
Raw traces of H-reflexes for FDI and ADM. Each trace is an individual trial that is representative of the group-averaged data of the H-reflex. **A**: Raw EMG traces of the H-reflex in FDI during preparation for 2^nd^ digit movement. **B**: Raw EMG traces of the H-reflex in FDI during preparation for 5^th^ digit movement. **C**: Raw EMG traces of the H-reflex in ADM during preparation for 2^nd^ digit movement. **D**: Raw EMG traces of the H-reflex in FDI during preparation for 5^th^ digit movement.

Experiment 2 examined whether spinal excitability was dependent on the muscle involved and the task being performed. The group mean F_max_ for the 2^nd^ digit was 23.7 N±10.7 and 15.5±6.8 for the 5^th^ digit. The repeated measures ANOVA revealed a significant 3-way interaction (*F*_(2,18)_ = 7.085, *p* = 0.005) across factors PHASE, MOVEMENT TYPE, and MUSCLE, and a significant main effect of PHASE (*F*_(2,18)_ = 13.198, *p*<0.001). *Figure 4* displays the group-averaged means (with standard error of the mean) for spinal excitability ratio during the pre-movement phases (PW1, PW2, PG) for each muscle (FDI, ADM) and each task (involved, uninvolved). The raw traces of the H-reflex for each movement condition are presented in *Figure 5*. Similar to the SAI data, post-hoc Tukey's test revealed effects in the PG phase. Spinal excitability was larger in FDI when it was involved versus uninvolved in the movement (*p*<0.05). Similarly, spinal excitability was larger in ADM when it was involved versus uninvolved in the movement (*p*<0.05). There were no muscle specific effects observed between FDI and ADM. In summary, these data indicate that spinal excitability was increased when each muscle was involved versus uninvolved in the task. Table 4 indicates the group means (with standard error of the mean) for all spinal excitability data.

**Table 4:** Spinal excitability data and spinal excitability ratio data for FDI and ADM during all conditions.

	Spinal Excitability Data							Spinal Excitability Ratio Data					
Muscle	Rest	Move 2^nd^ digit			Move 5^th^ digit			Move 2^nd^ digit			Move 5^th^ digit		
		PW1	PW2	PG	PW1	PW2	PG	PW1	PW2	PG	PW1	PW2	PG
FDI	0.01±0.003	0.02±0.003	0.02±0.003	0.03±0.009	0.02±0.004	0.02±0.005	0.02±0.007	1.07±0.088	1.16±0.144	1.82±0.288	0.04±0.003	1.16±0.144	1.24±0.158
ADM	0.06±0.016	0.05±0.014	0.04±0.007	0.06±0.015	0.06±0.020	0.04±0.010	0.09±0.020	1.11±0.126	0.87±0.171	1.19±0.167	1.05±0.139	0.90±0.121	1.69±0.215

Means for spinal excitability 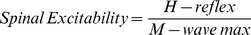 and spinal excitability ratio 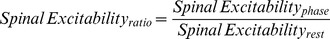 followed by standard error. HSD critical for a significance of 0.05 was calculated as 0.44 for comparison among spinal excitability ratio data.

## Discussion

The goal of the present study was to investigate SAI in muscles involved and uninvolved in a finger flexion task and determine whether the degree of SAI modulation depended on the specific digit. We chose to study two digits, the 2^nd^ and 5^th^, that contribute differently to whole hand function. Results indicated that SAI behaved differently in FDI compared to ADM. SAI in FDI was reduced when FDI was involved versus uninvolved in the task and this effect was observed only during movement initiation. In contrast, SAI in ADM was not modulated by its involvement in the task. Further, during movement initiation the reduction of SAI in FDI was greater compared to ADM when each muscle was involved in the task. In summary, SAI was modulated differently before movement onset for muscles controlling the 2^nd^ versus 5^th^ digit. The findings from this study are applicable to individuals with certain movement disorders and may provide insight into the direction of interventions for neurorehabilitation [9], [17], [19].

### Functional significance of SAI modulation

SAI creates a transient inhibition of M1 shortly after stimulation of a peripheral nerve and this inhibition might function to focus the neural activity in M1 during movement initiation. Similar to SAI, surround inhibition is a neurophysiological mechanism that inhibits surrounding muscle representations that are not performing the desired movement. Surround inhibition is most prominent during movement initiation [5], [17] and it is during this phase that we observed the most robust modulation of SAI across task and muscles. A previous report suggest that SAI may contribute to surround inhibition during EMG onset [8]. Our study extends these findings and indicates that SAI may contribute to surround inhibition even before EMG onset. When FDI was performing the task, there was the greatest reduction in SAI. When the FDI was uninvolved in the task, SAI remained intact. There was also was a trend for similar effects in ADM. These reductions in SAI when a muscle is involved in the task may be necessary to allow somatosensory input to increase activity in the area of M1 responsible for the desired motor output. When the muscle is uninvolved, SAI remains intact and may be necessary to prevent unwanted movements. Overall, the data suggests that SAI may function to inhibit or focus neural activity during movement initiation even before the onset of muscle activity and contribute to surround inhibition.

### Digit specific effects of SAI

SAI was modulated differently for SAI in FDI compared to ADM, which represents muscles controlling the 2^nd^ and 5^th^ digit, respectively. The 2^nd^ digit contributes more than the 5^th^ digit during static grip [12], gripping an object with varying force levels [13], and during different gripping tasks [10]. Amputation of the 2^nd^ digit results in a greater loss of overall hand function in relation to the 5^th^ digit [20]. Further, the cortical representation of the 2^nd^ digit may be larger than the 5^th^ digit [11], potentially because of its greater involvement in hand control [10], [12], [13]. In our study, the TMS stimulator output was greater for FDI in relation to ADM, likely because of the increased cortical representation of the 2^nd^ digit in relation to the 5^th^. This converging evidence indicates that the 2^nd^ digit plays a larger role in hand control. Since the 2^nd^ digit contributes more to hand function, one speculation is that this difference would allow a larger proportion of neurons representing the 2^nd^ digit within the primary somatosensory cortex (SI) to project to M1 and drive the greater modulation of SAI for a muscle controlling the 2^nd^ digit observed in this study.

We also observed reductions in SAI in FDI when it was uninvolved in the task and this could have been attributed to lack of isolation during 5^th^ digit movement. We chose finger flexion versus abduction/adduction movements to improve isolation of digit 2 and digit 5 movement, as reported elsewhere [17]. Even though our background EMG data indicated that there was no discernable activity in FDI during 5^th^ digit movement, it is possible that our techniques were unable to detect subthreshold depolarization of the alpha motorneurons directed toward FDI during this task. For example, during 5^th^ digit movement, subthreshold motor output to digits 2, 3 and 4 may be increased to provide hand stabilization or rapid recruitment if necessary. In the case of our data, it may be that during 5^th^ digit movement (i.e., FDI uninvolved), SAI in FDI is reduced because of the 2^nd^ digit's potential involvement in the task.

### Mechanisms of SAI modulation

To determine whether increases in spinal excitability may contribute to SAI modulation, H-reflexes were recorded since this technique recruits the same motorneuron pool as that recruited from a single TMS pulse [18]. During movement initiation, there was an increase in spinal excitability in the specific muscle involved in performing the task (see *Figure 4*), but this effect was not present during movement preparation. Therefore, during movement preparation, changes in SAI may be mediated by supraspinal mechanisms while changes in SAI during movement initiation appear to be mediated by both spinal and supraspinal mechanisms.

Although the supraspinal mechanisms that may reduce SAI during movement preparation and movement initiation are not well understood, several possibilities exist. One mechanism may involve an increase in GABAergic activity as GABA_A_ agonist lorazepam reduces SAI at rest [21]–[23]. The largest reduction of SAI in FDI during 2^nd^ digit movement may result from an interaction of GABAergic inhibitory interneurons or an interaction of different GABA_A_ sub-unit inhibitory interneurons, as previous work of these interactions has shown similar changes observed in our work [24], [25]. One other pathway for SAI modulation may involve the prefrontal cortex (PFC) and the thalamic reticular nucleus (TRN) [26], [27]. The PFC has dense connectivity with the TRN and this connectivity has the ability to modify sensory input via GABAergic inhibitory neurons in the thalamus and select inputs based on its relevancy to the task [26]. It is possible that PFC and TRN connectivity may modify the inputs reaching cortex and ultimately, modify SAI during movement preparation and initiation. Although speculative, our results provide ground work for future studies to explore this mechanism using pharmaceutical interventions that can alter GABAergic activity.

### Applications to movement disorders

In certain movement disorders such as focal hand dystonia (FHD), digit representations in SI overlap [28], [29]. Further in typically functioning adults, stimulation of multiple digits reduces the amount of inhibition within M1 in relation to single digit stimulation [30]. In FHD where digit representations overlap in SI, stimulation of a single digit during movement initiation could activate other digit representations in the cortex and cause a reduction in SAI across multiple muscles leading to unwanted movements of other digits. In FHD there is also lack of surround inhibition [5], [17] and maladaptive modulation of SAI may be adding to problems in this network. To support this statement, individuals with Parkinson's disease who also present with unwanted movements exhibit facilitation instead of SAI when a digit in the surrounding area is stimulated [31]. Further, a reduction in SAI is correlated with functional recovery from stroke with larger reductions in SAI being associated with more movement [32]. As a result, how SAI is modulated during movement initiation for muscles involved versus uninvolved in a task may be a marker for certain movement disorders that present with unwanted movements.

### Limitations

There are a few limitations that may impact our interpretation of the data. We observed differences in the modulation of SAI in FDI compared to ADM in a finger flexion task. One consideration is that such effects may have emerged because FDI may provide greater assistance to the long finger flexors compared to ADM. Future studies may examine the effects of SAI modulation in these two muscles during finger abduction, an action in which these muscles are primarily responsible for. We recorded H-reflexes to determine the level of spinal excitability in each muscle during the phases of movement. When measuring spinal excitability in FDI by stimulating the ulnar nerve, heteronymous excitation of the median nerve is possible [33] and could activate the first lumbrical muscle. A future study to test whether the type of nerve innervating the digit drives this SAI modulation may compare movements of a muscle in the thumb such as abductor pollicis brevis (i.e., median nerve) versus ADM (i.e., ulnar nerve). Further, we added light voluntary contraction when recording H-reflexes since this approach was necessary to record reflexes from these hand muscles and therefore, there was a small increase in force level to perform this task. Evidence in a study on lower limb spinal excitability, however, indicates that small increases in overall MVC does not affect H-reflex amplitude [34]. Thus, it is unlikely that the force level modification in the present work affected spinal excitability. Last, we elicited SAI in ADM and FDI with the same perceptual threshold to match the level of afferent input to the cortex. Our data indicates that SAI was greater in FDI compared to ADM at rest. It could be that the results of the study may have differed if SAI was matched for the same level of inhibition. However, the absolute magnitude of SAI for each muscle did not approach floor or ceiling levels thereby allowing SAI modulation to occur equally for each muscle tested. Further, since the opportunity for SAI modulation is similar for both muscles, the possible range of modulation is not limited or favoured for either muscle. If we had attempted to match SAI magnitude at rest across the two muscles, it is likely that the same effects would be observed, namely a very large reduction in FDI SAI. Therefore this limitation is unlikely to affect the overall interpretation of the data.

### Conclusion

SAI modulation prior to the onset of movement behaved differently for muscles controlling the 2^nd^ versus 5^th^ digit and how they differed depended on the movement phase tested. This work on the functionality of SAI has implications to individuals with certain movement disorders such as focal hand dystonia and Parkinson's disease that have difficulties preventing unwanted movements. Interventions aimed at improving SAI modulation during wanted and unwanted movements may improve hand function in certain movement disorders.
